# Comparison of C-MAC and McGrath-MAC Videolaryngoscopes for Intubation in Patients with Normal Airway by Donned Anaesthesiologists Using an Intubation Box During COVID-19 Pandemic: A Prospective, Randomized Study

**DOI:** 10.5152/TJAR.2021.21251

**Published:** 2022-08-01

**Authors:** Nishkarsh Gupta, Riniki Sarma, Saurabh Vig, Vinod Kumar, Anju Gupta, Seema Mishra, Sushma Bhatnagar

**Affiliations:** 1 Department of Onco-Anaesthesia and Palliative Medicine, All India Institute of Medical Sciences, New Delhi, India; 2 Department of Anaesthesia, Pain Medicine and Critical Care, All India Institute of Medical Sciences, New Delhi, India

**Keywords:** Aerosol, airway management, COVID-19, laryngoscopes, personal protective equipment, videolaryngoscopes

## Abstract

**Objective:**

Intubation is a highly aerosol-generating procedure. Recent airway management guidelines advocate the use of appropriate personal protective equipment, videolaryngoscope, and “intubation box” while intubating a suspected or infected coronavirus patient. We undertook a study to compare C-MAC videolaryngoscope with McGrath videolaryngoscope for tracheal intubation using an intubation box by donned anaesthesiologists.

**Methods:**

The patients were randomly allocated to 2 groups by computer-generated random numbers, depending upon the videolaryngoscope used. In group C, C-MAC videolaryngoscope (n = 30) was used, whereas McGrath videolaryngoscope was used in group M (n = 30). The primary outcome was the total time required for successful intubation. The secondary outcomes included the number of attempts required, Cormack and Lehane grade, the percentage of glottis opening score, the difficulty faced while using the device, and the user’s preference.

**Results:**

The time to intubation was 57.17 ± 19.98 seconds with C-MAC videolaryngoscope as compared to 57.93 ± 14.92 seconds with McGrath. Both the devices had a good percentage of glottis opening score. Twelve patients in each group were found to have a Cormack and Lehane grade of 1. The time to glottis visualization was more with McGrath than with C-MAC although not significant (23.8 ± 14.03 vs 20.10 ± 10.78 seconds). Both the devices were easy to use.

**Conclusions:**

Both C-MAC and McGrath videolaryngoscopes are equally effective devices for intubation by a donned anaesthesiologist using an intubation box. McGrath with a disposable blade should be preferred for intubation in these conditions.

## Main Points

- Videolaryngoscopes (VL) have become an integral part of self-protective precaution while intubating a patient nowadays.
- The use of an intubation box further acts as a barrier against aerosol exposure.
- Our study compared C-MAC and McGrath VL for intubation using an intubation box by an anaesthesiologist donned in full personal protective equipment.
- Both C-MAC and McGrath VLs are equally effective devices for intubation in the above scenario.
- Out of the 2, McGrath VL with a disposable blade should be preferred.

## Introduction

Intubation is a highly aerosol-generating procedure.^1^ In the setting of the coronavirus disease 19 (COVID-19) outbreak, healthcare providers taking care of critically ill patients need to perform the best practices of intubation and ventilation along with self-protective precautions. Airway management guidelines suggest the use of appropriate personal protective equipment (PPE) and the use of videolaryngoscopes (VLs) for intubating a COVID patient.^2,3^ The use of a VL makes intubation easier and minimizes exposure by increasing the distance between the patient and the intubating anaesthesiologists. Literature also advocates the use of protective equipment like transparent fiberglass “intubation or aerosol box” to cover the patient's head for the prevention of the spread of aerosols during intubation.^4^ However, a combination of eye shields, a well-fitted N-95 mask, and an external transparent visor used to protect the face from aerosols may lead to hampered vision due to fogging of eye shields and poorly fitted equipment. Also, using an “aerosol box” for additional protection may further restrict hand movement and may pose additional difficulties in successful intubation.

Since there are several VLs, there is a need to ascertain the best among them to use in these scenarios. No study has compared the performance of C-MAC VL and McGrath VL for tracheal intubation using an intubation box by anaesthesiologists donned in the PPE. Hence, we decided to find out the better VL among the 2 with the primary outcome being the time required for successful intubation.

## Methods

After Institutional review board approval and ethical clearance (IEC-407/08.05.2020), this prospective, randomized study was conducted on patients undergoing surgery under general anaesthesia in a public tertiary care hospital. The study was prospectively registered in Clinical Trials Registry-India (CTRI), and the registration number was CTRI/2020/05/025489. A written informed consent was taken from all the patients.

**Figure 1.**
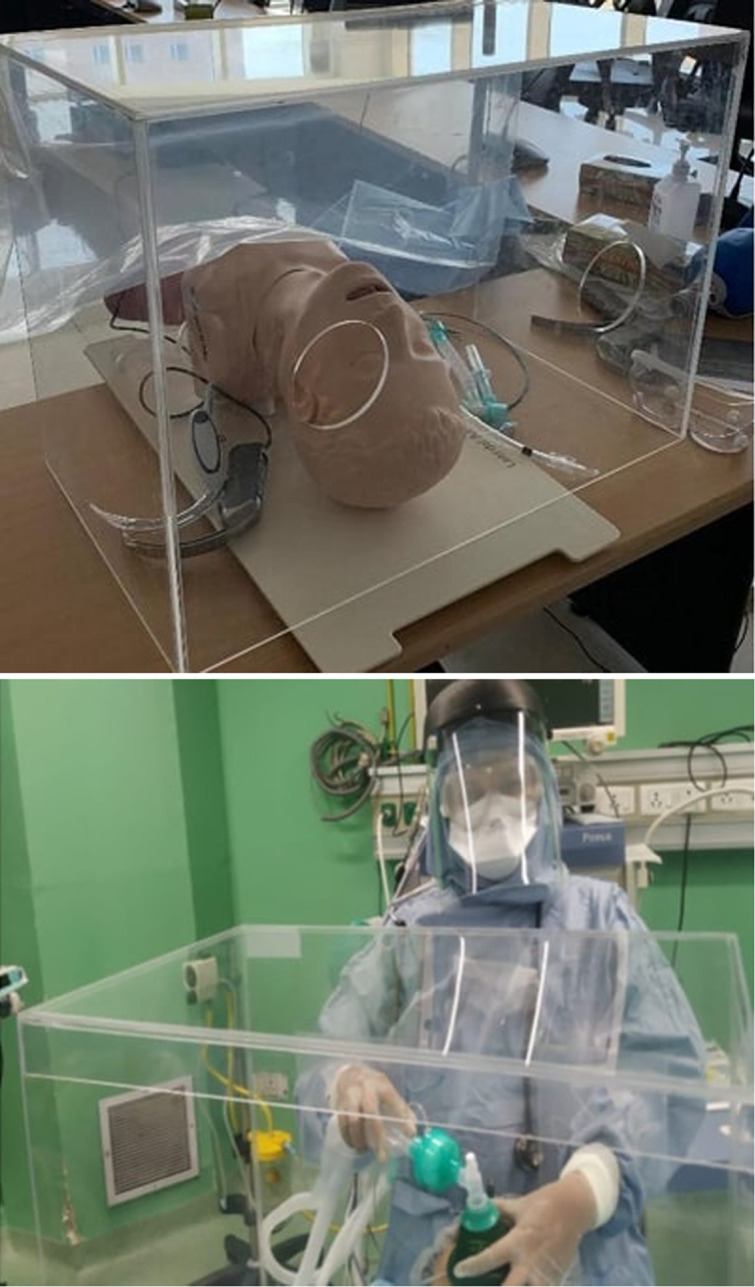
Intubation box used in our study.

The inclusion criteria for the study were patients aged 18-70 years, American Society of Anesthesiologists (ASA) I/II undergoing elective general surgery. We excluded patients with the presence of predictors of the difficult bag and mask ventilation (presence of beard, body mass index > 35, snoring, edentulous, intraoral tumors, receding chin, etc.) and intubation, including decreased inter-incisor distance (<2 cm), short thyromental distance (<6 cm), and reduced neck extension (<80° from neck flexion), cervical spine instability, or risk of pulmonary aspiration. A detailed pre-anaesthetic check-up was conducted, and investigations were done as per the age, surgical condition, and associated disease of patients. Those anaesthesiologists who have done a minimum of 50 intubations with both VLs and practiced intubation through the intubation box at least 10 times were allowed to participate in the study. The intubation box was cuboidal in shape made of transparent fiberglass with the following dimensions: base and top 70 × 40 cm, front 70 × 50 cm, lateral 50 × 40 cm, and the back covered with a transparent polythene sheet. It had 2 circular channels of 10 cm diameter on the front side as working channels (Figure 1).

The patients were randomly allocated to 2 groups by computer-generated random numbers, depending upon whether a C-MAC VL (Karl Storz, Tuttlingen, Germany) was used (group C, n = 30) or McGrath VL (Aircraft Medical Ltd, Edinburgh, UK) was used (group M, n = 30). The group allocation was concealed in opaque envelopes.

In the operation theater, the patients were attached to standard monitors (echocardiogram, non-invasive blood pressure, and oxygen saturation) and an intravenous line was established. Injection fentanyl 2 μg kg-1 was given intravenously to all the patients 5 minutes before the procedure. Anaesthesia was induced with injection propofol 2 mg kg-1 and muscle relaxants rocuronium 1.2 mg kg-1 given after mask ventilation. After 1 minute of ventilation with O_2,_ orotracheal intubation was attempted with an appropriate size styleted endotracheal tube (ETT) (size, 7.5 in males and 7.0 in females) by VL as per group allocation. The need for additional maneuvres like optimum external laryngeal manipulation and head manipulation was noted. The position of ETT was confirmed with a square wave capnograph.

The primary outcome of our study was the total time required for successful intubation (total time from passage of the device into the oral cavity till the first appearance of regular capnograph waveform). The secondary outcomes included the number of attempts required for successful intubation, Cormack and Lehane (CL) grade, percentage of glottic view seen (POGO), optimization maneuvres needed for best glottic view, the difficulty faced while using the device (graded on a scale of 0-10; 0: extremely easy and 10: extremely difficult), and oropharyngeal morbidity like dental trauma, mucosal bleeding, etc. We also assessed the user’s preference between the 2 laryngoscopes based on the ease of insertion of the laryngoscope blade, ease of visualization of vocal cords, ease of passing the endotracheal tube, and overall preference.

### Sample Size Estimation

There was no study on this scenario reported earlier. We did a pilot study on a mannequin using C-MAC and McGrath. The mean time required for successful tracheal intubation was 26 seconds and 33 seconds with a standard deviation of 8.5 seconds and 9.4 seconds, respectively. With a clinically important difference of 7 seconds for the same, 5% level of significance, and 80% power, a sample size of 26 was calculated. We decided to do 30 patients in each group to factor in the dropouts.

### Statistical Analysis

Statistical analysis was performed using Statistical Package for the Social Sciences Version 24 (IBM Corp.; Armonk, NY, USA). The normal distribution of data was tested using the Shapiro–Wilk test. Comparison of success rates was analyzed using chi-squared tests. Analyses of continuous data were performed using the Student’s *t*-test (unpaired) (for parametric data) and independent samples Mann–Whitney *U* test (for non-parametric data) with Bonferroni correction. *P* < .05 was considered significant.

## Results

**Figure 2.**
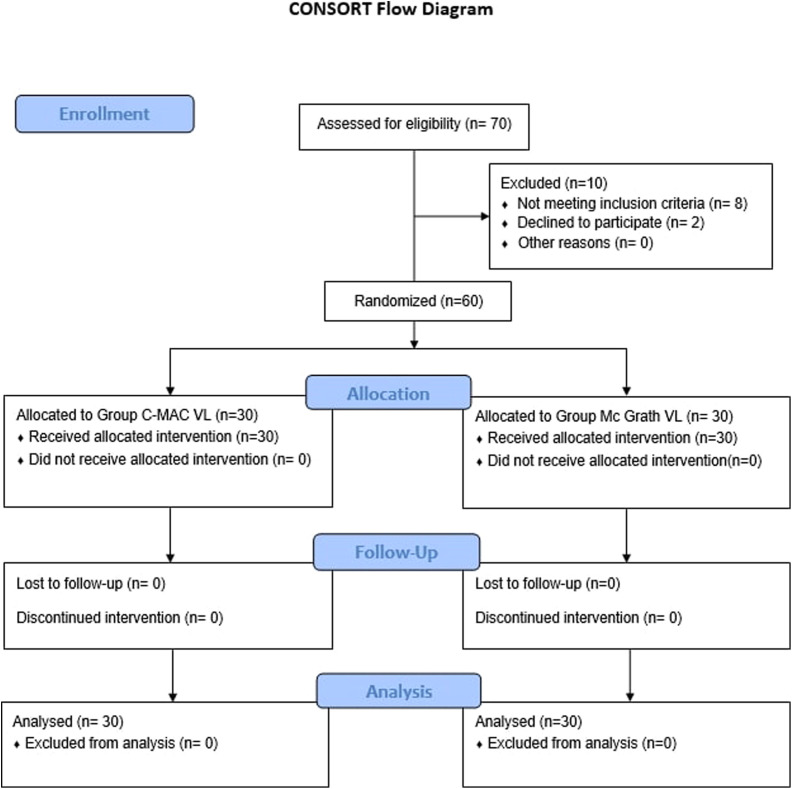
CONsolidated Standards of Reporting Trials (CONSORT) diagram.

A total of 70 patients were screened for eligibility, of which 8 did not meet inclusion criteria and 2 declined to participate. The remaining 60 patients were randomized into 2 groups of 30 each (Figure 2). Both the groups were similar in age, gender weight, ASA status, and airway parameters like modified Mallampati class (Table 1). The POGO score and CL grade were comparable. Both the devices had a good POGO score. Twelve patients in each group were found to have a CL grade of 1. The time to glottis visualization was more with McGrath than with C-MAC although not significant (23.8 ± 14.03 vs 20.10 ± 10.78 seconds). The time to intubation was 57.17 ± 19.98 seconds with C-MAC VL and 57.93 ± 14.92 seconds with McGrath VL (Table 2). Ease of blade insertion was rated easy in 19 cases for C-MAC and 16 cases for McGrath. Similarly, there was no significant difference in the Numerical Rating Scale (NRS) scales of ease of obtaining glottis views, ease of bringing ETT into glottis, or passing the ETT to trachea from glottis. The median (range) Visual Analog Scale (VAS) of ease of use of these devices was 2.^1-8^ The first attempt success rate was 90% with C-MAC and 80% with McGrath (*P*  = .254). All the cases that failed the first intubation attempt were intubated successfully in the second attempt with McGrath, whereas 1 case of C-MAC required third attempt. The need for optimization maneuvre was similar with the 2 devices (*P*  = .756) (Table 3). There was no associated oropharyngeal morbidity like mucosal bleeding or dental trauma in any of the cases. Hemodynamic changes occurring during intubation were also insignificant between the 2 groups (Table 4).

## Discussion

Videolaryngoscopes have gained popularity since the advent of COVID-19, and in the past few years, VLs have become an integral component of various airway guidelines.^5,6^ The main advantage of a VL over a direct laryngoscope is that we no longer need to align the oral, laryngeal, and pharyngeal axes. The camera present on the tip of the VL directs the glottis view to a large monitor that makes looking into the oral cavity unnecessarily. Thus, in the case of a COVID-19 scenario, VLs can lessen the exposure to aerosols. This exposure can further be reduced by using an “intubation box” or “aerosol box.” The intubation box is designed to be placed over a patients’ head during an aerosol-generating procedure such as intubation and has 2 holes for the arms of the anaesthetist to manage the airway. Studies have shown that the use of an intubation box increases the time to intubate.^7^ But since this box decreases the risk of exposure to aerosols, it should be used routinely in all suspected or COVID-19-infected patients. Hence, we decided to use 2 VLs for the comparison of tracheal intubation with an intubation box. We chose C-MAC and McGrath VLs as these are the 2 most used devices in our institution.

C-MAC VL is of appropriate use with shorter intubation times in various studies as compared to Macintosh direct laryngoscope.^8,9^ McGrath-MAC has a slimmer blade that is disposable with a liquid crystal diplay screen attached to the handle. It is lighter and more compact as compared to the C-MAC VL.^10^ There have been various studies comparing C-MAC and McGrath VLs. Our study showed that there was no significant difference in the time taken to intubate by both the VLs. A study done by Shin et al^11^ in McGrath and C-MAC showed that the time taken to intubate between the VLs was comparable and significantly better than a direct laryngoscope in patients with a normal airway. The participants of this study also chose the McGrath blade as more useful than the other 2. In another study,^12^ although McGrath VL was found to provide more grade 1 laryngoscopic views, it required longer intubation times and more attempts than C-MAC VL. The participants in this study also rated C-MAC VL as being easier to use. They did not report any significant change in the number of successful intubations and complications. Similarly, in our study, we found that 4 participants found the use of the C-MAC blade extremely easy, whereas only 1 reported McGrath blade to be extremely easy to use. McGrath VL has been found to provide a good laryngeal view in various other studies including manikins and patients with difficult airways, but a good laryngeal view does not always translate into easy intubation.^13,14^ This difference between good vision and the relatively poorer intubation success rate is known with VL and could be further explained by the extra layer of vision obtundation by the aerosol box.

There have been many studies on intubation boxes to demonstrate the time to intubation and intubation success rates.^7^ ^,15^ ^,16^ Begley et al^15^ study showed that intubation boxes increased the time to intubate and thus increased the chances of hypoxia in patients. Another study^[Bibr b7-tjar-50-4-255]^ found that in difficult airway scenarios, the use of an intubation box increased the time to intubation by 7 seconds.^7^ They also found that there was an increased need for optimization maneuvres, intubation attempts, and failed attempts. In our study, one-third of the patients required an optimization maneuvre in both groups. Another meta-analysis by Lim et al^16^ found that the time to intubate was increased with the use of an intubation box, but it was relatively shorter when intubation was performed by an experienced anaesthesiologist using VL. In our study since aerosol box was used with both VLs the time to intubation did not differ significantly. Similarly, the same is true for other parameters like the number of attempts for successful intubation, ease of use of VLs, ease of visualizing glottis, or passing ETT into the glottis. We would attribute the results to the experience level of our participants. Perhaps, the inclusion of participants with lesser experience could yield a different result.

Choosing the right VL is important in a suspected COVID scenario as these patients requiring intubation are more prone to desaturation. Ideally, the laryngoscope should be easy to use, requiring minimum time for intubation without many complications. The C-MAC VL has a bulky design, needs disinfection under specific protocols, and is cumbersome for transportation. Hence considering the results of our study showing similar intubation times for orotracheal intubation of adults with 2 VLs, we would recommend McGrath VL for routine use in the COVID-19 pandemic due to inherent advantages of McGrath VL (disposable blades, portable design, and reduced initial cost).

There are a few limitations to this study. We were unable to blind the operating anaesthesiologists to the type of laryngoscope to be used. This could lead to bias if the anaesthetist already preferred 1 particular VL as per randomization. However, the operator criteria set would have nullified the bias. The sample size was low, and a larger sample size may result in different results. We did not include patients with anticipated difficult airway in our study as results may be different in such cases.

## Conclusion

Both C-MAC and McGrath VLs are equally effective devices for intubation by a donned anaesthesiologist using an intubation box. We would suggest that McGrath VL with a disposable blade design is better suited for intubation in patients during the pandemic with highly infectious diseases like the present COVID-19 scenario and should be chosen for intubation in adults with normal airways.

## Figures and Tables

**Table 1.** Baseline Characteristics

	C-MAC	McGrath	*P*
Age (years), mean (SD)	46.9 (7.90)	47.77 (8.57)	.657
Weight (kg), mean (SD)	56.77 (6.43)	60.81 (5.60)	.947
Gender (male/female)	18/12	16/14	.301
ASA (1/2)	11/19	10/20	.787
Modified Mallampati class (1/2/3/4)	8/10/11/1	10/12/6/2	.530

SD, standard deviation.

**Table 2.** Time Taken for Glottis Visualization and Intubation

	C-MAC	McGrath	*P*
POGO, mean (SD)	76.90 (18.98)	76.53 (17.156)	.712
CL grade 1/2a/2b	12/11/7	12/13/5	.425
Time to glottic view (seconds), mean (SD)	20.10 (10.78)	23.8 (14.03)	.615
Time to intubation (seconds), mean (SD)	57.17 (19.98)	57.93 (14.92)	.134

POGO, percentage of glottis opening score; CL, Cormack and Lehane grade; SD, standard deviation.

**Table 3.** Users’ Preference Regarding the 2 Videolaryngoscopes

Variables	C-MAC	McGrath	*P*
Ease of blade insertion (very easy/easy/neutral/hard/very hard)	4/19/7/0/0	1/16/11/2/0	.162
Ease of obtaining glottis visualization (very easy/easy/neutral/hard/very hard)	2/17/10/1/0	1/18/10/1/0	1.000
Ease of bringing ETT to glottis (very easy/easy/neutral/hard/very hard)	1/14/9/5/1	0/18/12/0/0	.063
Ease of passing ETT to trachea (very easy/easy/neutral/hard/very hard)	2/14/10/2/2	2/15/13/0/0	.396
Number of attempts (1/2/3)	27/2/1	24/6/0	.254
Optimization maneuvers (none/1 used/2 used)	18/11/1	19/9/2	.756
VAS score (0-10), median (IQR)	2 (1-8)	2 (1-8)	.914

ETT, endotracheal tube; IQR, interquartile range.

**Table 4.** Hemodynamic Changes

Time	C-MAC	McGrath	*P*
**Mean arterial pressure** (mm Hg), mean (SD)			
Baseline	86.40 (13.52)	88.54 (13.18)	.529
1 minute after intubation	87.50 (14.78)	87.04 (11.28)	.162
5 minutes after intubation	82.43 (13.54)	85.39 (12.99)	.599
15 minutes after intubation	84.93 (12.32)	84.11 (12.24)	.733
30 minutes after intubation	85.57 (12.59)	85.04 (12.39)	.554
**Pulse rate** (pulse min-1), mean (SD)			
Baseline	85.53 (15.69)	82.93 (12.40)	.141
1 minute after intubation	88.57 (17.90)	82.86 (14.69)	.433
5 minutes after intubation	83.20 (16.17)	83.50 (14.32)	.625
15 minutes after intubation	81.30 (14.03)	81.43 (12.39)	.577
30 minutes after intubation	80.80 (12.67)	79.54 (12.72)	.745

SD, standard deviation.
